# Analysis of maternal genetic structure of mitochondrial DNA control region from Tai-Kadai-speaking Buyei population in southwestern China

**DOI:** 10.1186/s12864-023-09941-x

**Published:** 2024-01-11

**Authors:** Yuhang Feng, Li Chen, Xiaoxue Wang, Hongling Zhang, Qiyan Wang, Yubo Liu, Xiaoye Jin, Meiqing Yang, Jiang Huang, Zheng Ren

**Affiliations:** Department of Forensic Medicine, Guizhou Medical University, Guiyang, 550004 Guizhou China

**Keywords:** mtDNA, Control region, Guizhou Buyei, Haplotype, Haplogroup

## Abstract

**Background:**

Even though the Buyei are a recognised ethnic group in southwestern China, there hasn’t been much work done on forensic population genetics, notably using mitochondrial DNA. The sequences and haplogroups of mitochondrial DNA control regions of the Buyei peoples were studied to provide support for the establishment of a reference database for forensic DNA analysis in East Asia.

**Methods and results:**

The mitochondrial DNA control region sequences of 200 Buyei individuals in Guizhou were investigated. The haplotype frequencies and haplogroup distribution of the Buyei nationality in Guizhou were calculated. At the same time, the paired *Fst* values of the study population and other populations around the world were computed, to explore their genetic polymorphism and population relationship. A total of 179 haplotypes were detected in the Buyei population, with frequencies of 0.005–0.015. All haplotypes were assigned to 89 different haplogroups. The haplotype diversity and random matching probability were 0.999283 and 0.0063, respectively. The paired *Fst* genetic distances and correlation p-values among the 54 populations revealed that the Guizhou Buyei was most closely related to the Henan Han and the Guizhou Miao, and closer to the Hazara population in Pakistan and the Chiang Mai population.

**Conclusions:**

The study of mitochondrial DNA based on the maternal genetic structure of the Buyei nationality in Guizhou will benefit the establishment of an East Asian forensic DNA reference database and provide a reference for anthropological research in the future.

**Supplementary Information:**

The online version contains supplementary material available at 10.1186/s12864-023-09941-x.

## Introduction

Human mitochondrial DNA (mtDNA) has an elevated copy number in comparison with nuclear DNA, so it can be used as a forensic sample if nuclear DNA is not available [1]. In addition to its elevated copy number, mtDNA is advantageous in that it does not recombine, and mutations accumulate over time. Matrilineal pedigree analysis can be performed based on mtDNA sequences if sufficient autosomal DNA is not available [2]. With the application of numerous molecular biological detection techniques in medicine, mtDNA sequence analysis has been properly verified and has become a reliable technique for detecting biological evidence in forensic criminal cases.

Buyei people are one of 17 permanent ethnic minorities in southwest China’s Guizhou Province, descended from the ancient “Baiyue”, mainly distributed in Guizhou, Yunnan, Sichuan, and other provinces, of which Guizhou Province has the largest population, accounting for 97% of the nationwide Buyei population. The Buyei language is part of the Tai-Kadai family of Sino-Tibetan languages. (https://www.britannica.com/topic/Buyei) With its lengthy history and distinctive customs, the Buyei ethnic group deserves anthropological and demographic genetic studies. Currently, there is a lack of mtDNA sequence data for the Buyei people, which is insufficient for forensic science and demographic genetic studies. In our study, the mtDNA control region sequences of 200 unrelated individuals of the Buyei people of Guizhou Province were analyzed to aid in the establishment of the database and the determination of ancestral composition from the point of view of matrilineal inheritance.

## Materials and methods

### Sample collection

Blood samples were collected from 200 unrelated paternity tests of Buyei individuals in Guizhou Province. All of these people are indigenous Buyei people of Guizhou who are not related by blood within three generations. All participants provided written informed consent after we explained the purpose and procedure of the study. The mitochondrial DNA control region sequences of all Tai-Kadai-speaking Buyei individuals generated in this study have been submitted to GenBank (http://www.ncbi.nlm.nih.gov/BankIt/), and the accession numbers are ON983171-ON983370.

### DNA extraction, amplification, and sequencing

In accordance with the manufacturer's instructions, DNA was extracted using the QIAamp DNA Mini kit (Qiagen, Hagen, Germany). GeneAmp PCR System 9700 (Thermo Fisher, Waltham, MA) was used to amplify the entire mtDNA control region using primers F15975 and R637 (displayed in Table S1; in the supplementary material). Each reaction mixture contained One-Shot LA PCR Mix 25 μl (TaKaRa Bio Inc., Dalian, China), 0.4 μM of each primer, and 10 ng DNA. The amplification was carried out under the conditions of 95℃ for 5 min, 30 amplification cycles of 94℃ for 30 s, 60℃ for 30 s, and 72℃ for 30 s, and one full extension cycle at 72℃ for 10 min. Purification of the PCR products was performed using Exonuclease I (TaKaRa Bio Inc., Dalian, China) and Shrimp Alkaline Phosphatase (TaKaRa Bio Inc., Dalian, China), and they were sequenced with the BigDye^TM^ Terminator version 3.0 Ready Reaction Cycle Sequencing Kit (Thermo Fisher, Waltham, MA) using the 3730xl DNA Analyser (Thermo Fisher Scientific, Waltham, MA) according to the manufacturer’s manual. Following an earlier report, sequencing primers were used [3]. A combination of forward and reverse-direction sequencing was used to enhance the data's accuracy.

### Sequence nomenclature and haplogroup assignment

With the help of the DNAman software (http://www.lynnon.com/), forward and reverse sequences were aligned and compared with the revised Cambridge Reference Sequences (rCRSs) [4]. As suggested by Parson et al., insertions at nt16193, nt309, nt315, and nt573 were omitted from statistical analyses and all comparisons [5]. Haplogroup assignment was performed using PhyloTree Build 17 [6]-based Haplogrep [7] and EMMA [8].

### Statistical analysis

The direct counting method was used to calculate haplotype and haplogroup frequencies. The diversity of haplotype and random matching probability were estimated according to Stoneking et al [9]. In addition, our data are compared with other data available from the literature, including Guizhou Miao [10], Henan Han [11], Chinese Bai [12], Xinjiang Mongolian [13], Southwest Gelao [14], Yunnan Dai [15], Liaoning Han [15], Pinghua Han in Guangxi [16], Xinjiang Kazakh [17], Beijing Han [15], Mulao in Guangxi [18], Chinese Hui [19], Kashmiri [20], Hazara people of Pakistan [21], African Americans in Orange, California [22], African Americans in Vermont, California [22], Parana of Brazil [23], Iranian [24], Alto Parana [25], Iraqi [26], Arabian [26], Kuwaiti [26], Palestinian [26], Anatolians in Turkey [26], Kurds in Iran [27], Bosnian of Roma [28], Gulagic in Africa [29], Punjab in Pakistan [30], Sierra Leone Mendes in West Africa [29], Tengnai in Sierra Leone [29], Mandinka in Sierra Leone [29], Polish Gypsies [31], Finns [32], Turks [33], Northern Tunis [34], North-central Moroccan [34], Mozabit [34], Moroccan Berber [34], Gdansk in northern Poland [35], Upper Silesia in southern Poland [35], Novgorod in northwest Russia [35], Christmas Island in Australia [36], Romanians [37], Pukhtunhwa in Pakistan [38], Mosuo [39], Bengali [40], Khattak of the Peshawar Valley [41], Kheshgi of the Peshawar Valley [41], Slovak [42], Hokkaido in Japan [43], Vietnamese [44], Dutch [45] and People from Chiang Mai, Thailand [46]. The paired *Fst* values were computed with Arlequin version 3.5 software [47], and the *Fst* matrix data was imported into the “pheatmap” package of R software (https://www.r-project.org/) to plot the heatmap. To gain a more comprehensive understanding of the population relationships among various populations, the principal component analysis (PCA) was conducted based on haplogroup frequencies using the Multivariate Statistical Package version 3.22 (MVSP) [48]. The neighbor-joining (NJ) phylogenetic tree based on the pairwise *Fst* value matrix was constructed with the assistance of MEGA 11 software [49].

## Results

In 200 individuals, a total of 179 haplotypes (89.5%) were observed (Table S1, in the supplementary material), of which 163 haplotypes were unique (91.1%). The most common haplotypes were 16140C, 16183C, 16188.1C, 16189C, 16266A, 16519C, 73G, 210G, 263G, 309.1C, 315.1C, 522DEL, 523DEL (haplogroup B5a); 16108 T, 16129A, 16162G, 16172C, 16304C, 73G, 150 T, 195C, 248DEL, 263G, 315.1C, 522DEL, 523DEL (haplogroup F1a1a1); 16129A, 16192 T, 16223 T, 16297C, 73G, 150 T, 182 T, 199C, 263G, 315.1C, 489C (haplogroup M7b1a1 + (16192)); 16129A, 16192 T, 16223 T, 16297C, 73G, 150 T, 199C, 263G, 309.1C, 315.1C, 489C (haplogroup M7b1a1 + (16192)); 16086C, 16297C, 16324C, 16399G, 73G, 199C, 263G, 315.1C, 489C (haplogroup M7b1a2a), that were all shared by three individuals (1.5%). The haplotype diversity and random matching probability of the mtDNA control region of the Buyei nationality in Guizhou were 0.999283 and 0.0063, respectively.

All samples were assigned to 89 different haplogroups and sub-haplogroups (Table 1, Figure S1 in the Supplementary Materials). The most common haplogroups were B5a (18 cases, 9%), followed by M7b1a1 + (16192) (17 cases, 8.5%), B4b1 (7 cases, 3.5%), and R9 (14 cases, 7%). At the broader haplogroup level, the most common is M (69 samples, 34.5%), followed by B (41 samples, 20.5%), and F (33 samples, 16.5%).

**Table 1:** Haplogroup frequencies of 200 Chinese Buyei individuals

Broad Haplogroup	Number	Frequency	Haplogroup	Number	Frequency
A	2	0.01	A15	1	0.005
-	-	-	A19	1	0.005
B	41	0.205	B4	1	0.005
-	-	-	B4 + 16261	4	0.02
-	-	-	B4a1a	1	0.005
-	-	-	B4a1c	1	0.005
-	-	-	B4a4	1	0.005
-	-	-	B4b1	1	0.005
-	-	-	B4b1a + 207	1	0.005
-	-	-	B4b1a2a	1	0.005
-	-	-	B4c1b	1	0.005
-	-	-	B4c1b2b	1	0.005
-	-	-	B4c1b2c1	1	0.005
-	-	-	B4c2	1	0.005
-	-	-	B4g	2	0.01
-	-	-	B4h1	1	0.005
-	-	-	B4k	1	0.005
-	-	-	B4m	1	0.005
-	-	-	B5a	18	0.09
-	-	-	B5a1d	1	0.005
-	-	-	B5a2	1	0.005
-	-	-	B5b	1	0.005
C	9	0.045	C	1	0.005
-	-	-	C4a1	1	0.005
-	-	-	C4c1b	6	0.03
-	-	-	C7 + 16051	1	0.005
D	13	0.065	D4a3	3	0.015
-	-	-	D4a7	1	0.005
-	-	-	D4b2b	1	0.005
-	-	-	D4b2b2a	1	0.005
-	-	-	D4e1a	3	0.015
-	-	-	D4g2a	1	0.005
-	-	-	D5b	2	0.01
-	-	-	D6c	1	0.005
F	33	0.165	F1a1	4	0.02
-	-	-	F1a1a	2	0.01
-	-	-	F1a1a1	3	0.015
-	-	-	F1a1d	4	0.02
-	-	-	F1a3a2	1	0.005
-	-	-	F1a4a	1	0.005
-	-	-	F1c1a1	1	0.005
-	-	-	F2b1	3	0.015
-	-	-	F2d	3	0.015
-	-	-	F3a + 207	4	0.02
-	-	-	F3a1	5	0.025
-	-	-	F3b1a2	1	0.005
-	-	-	F4a	1	0.005
G	5	0.025	G2a	2	0.01
-	-	-	G2a + 152	1	0.005
-	-	-	G2b1a1	1	0.005
-	-	-	G3	1	0.005
M	69	0.345	M	1	0.005
-	-	-	M1	1	0.005
-	-	-	M11 + 200	1	0.005
-	-	-	M11a	1	0.005
-	-	-	M12	1	0.005
-	-	-	M1a3	1	0.005
-	-	-	M33a1b	2	0.01
-	-	-	M74a	3	0.015
-	-	-	M76a	1	0.005
-	-	-	M7b1a1	2	0.01
-	-	-	M7b1a1 + (16192)	17	0.085
-	-	-	M7b1a1a	9	0.045
-	-	-	M7b1a1a3	1	0.005
-	-	-	M7b1a1b	8	0.04
-	-	-	M7b1a1e2	1	0.005
-	-	-	M7b1a2a	3	0.015
-	-	-	M7c1c2	4	0.02
-	-	-	M7c1c2a	1	0.005
-	-	-	M8a	1	0.005
-	-	-	M8a2′3	2	0.01
-	-	-	M8a2a	2	0.01
-	-	-	M8a3a	1	0.005
-	-	-	M9a1a	1	0.005
-	-	-	M9a1a1c	1	0.005
-	-	-	M9a1a2*2	1	0.005
-	-	-	M9a1b + 150	1	0.005
-	-	-	M9a'b	1	0.005
N	4	0.02	N5	1	0.005
-	-	-	N9a1	1	0.005
-	-	-	N9a10 + 16311	2	0.01
R	24	0.12	R9	14	0.07
-	-	-	R9b1	3	0.015
-	-	-	R9b1a3	2	0.01
-	-	-	R9b1b	1	0.005
-	-	-	R9b2	1	0.005
-	-	-	R9c1	1	0.005
-	-	-	R9c1a	1	0.005
-	-	-	R9c1b1	1	0.005

All samples were assigned to 89 different haplogroups and sub-haplogroups (Figure S1 in the Supplementary Materials). The most common haplogroups were B5a (18 cases, 9%), followed by M7b1a1 + (16192) (17 cases, 8.5%), B4b1 (7 cases, 3.5%), and R9 (14 cases, 7%). At the broader haplogroup level, the most common is M (69 samples, 34.5%), followed by B (41 samples, 20.5%), and F (33 samples, 16.5%)

**Fig. 1:**
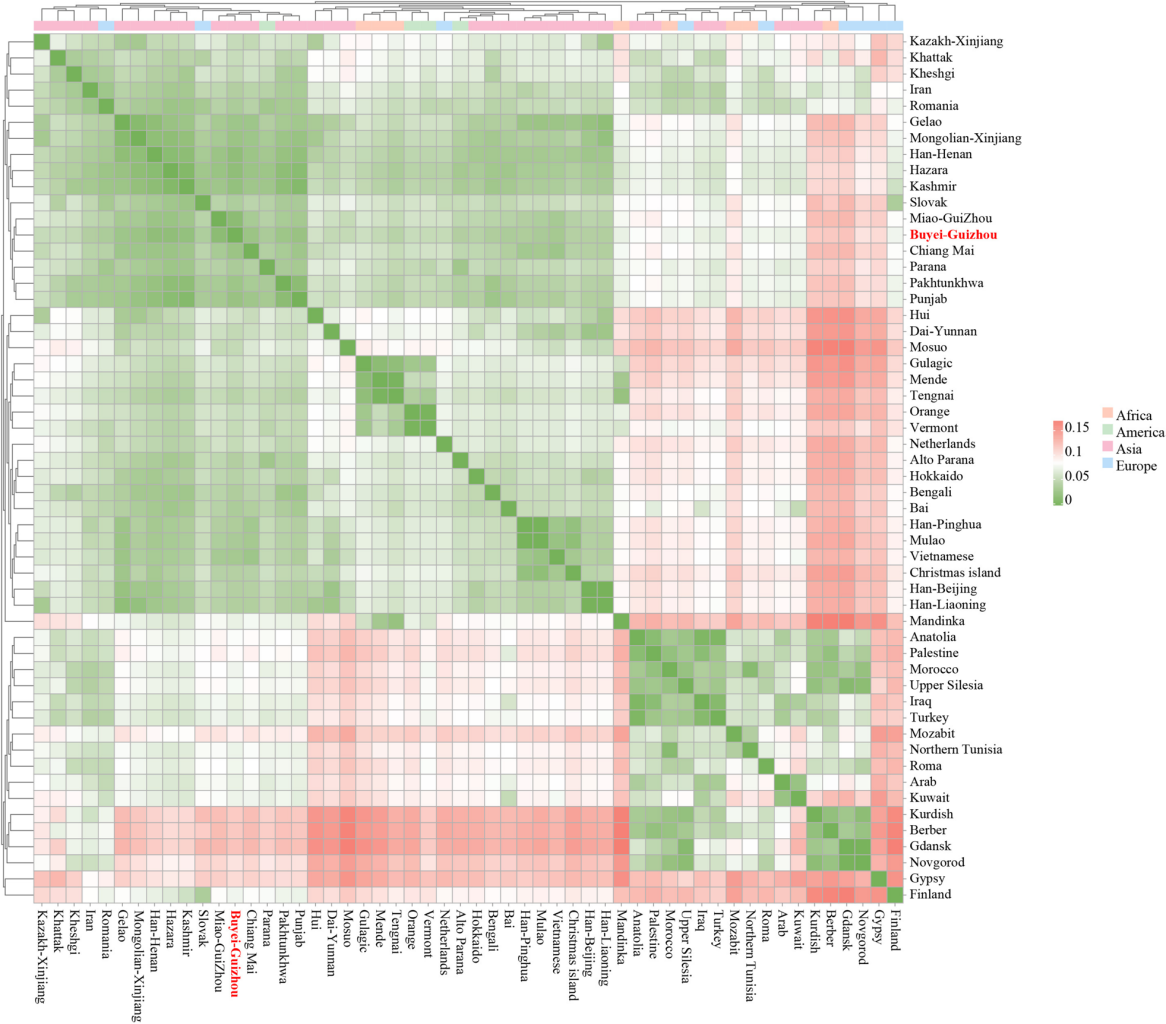
Heatmap for genetic distances between the studied Guizhou Buyei population and 53 worldwide reference populations. Visualising the *Fst* values with different colours. The powder orange represents the high *Fst* values, and green represents the low *Fst* values. Highlight the Guizhou Buyei population in red font

In order to further reveal the genetic similarities and divergences among the Guizhou Buyei population and 53 reference worldwide populations, the paired *Fst* genetic distance between the studied population and reference populations was calculated. The results were displayed in the form of a heatmap (Fig. 1). As shown in Table S2 of the supplementary material, the paired *Fst* and p-values of the Guizhou Buyei population and 53 other published populations worldwide were calculated. Among the 13 reference populations in China, the genetic differentiation between the Guizhou Buyei and Guizhou Miao was the smallest (with the closest genetic affinity, *Fst* = 0.01508), followed by the Henan Han population (*Fst* = 0.01799). The genetic distance between the northwest Hui and Guizhou Buyei was the largest (with the farthest genetic affinity, *Fst* = 0.05908). The showed that among 40 global reference populations (except China), the Guizhou Buyei population and Pakistan Hazara population had the smallest genetic distance (with the closest genetic affinity, *Fst* = 0.01783), followed by the Kashmiri (*Fst* = 0.02084), and had the largest genetic differentiation (with the farthest genetic affinity, *Fst* = 0.12165) with the Gdansk people in Poland.

**Fig. 2:**
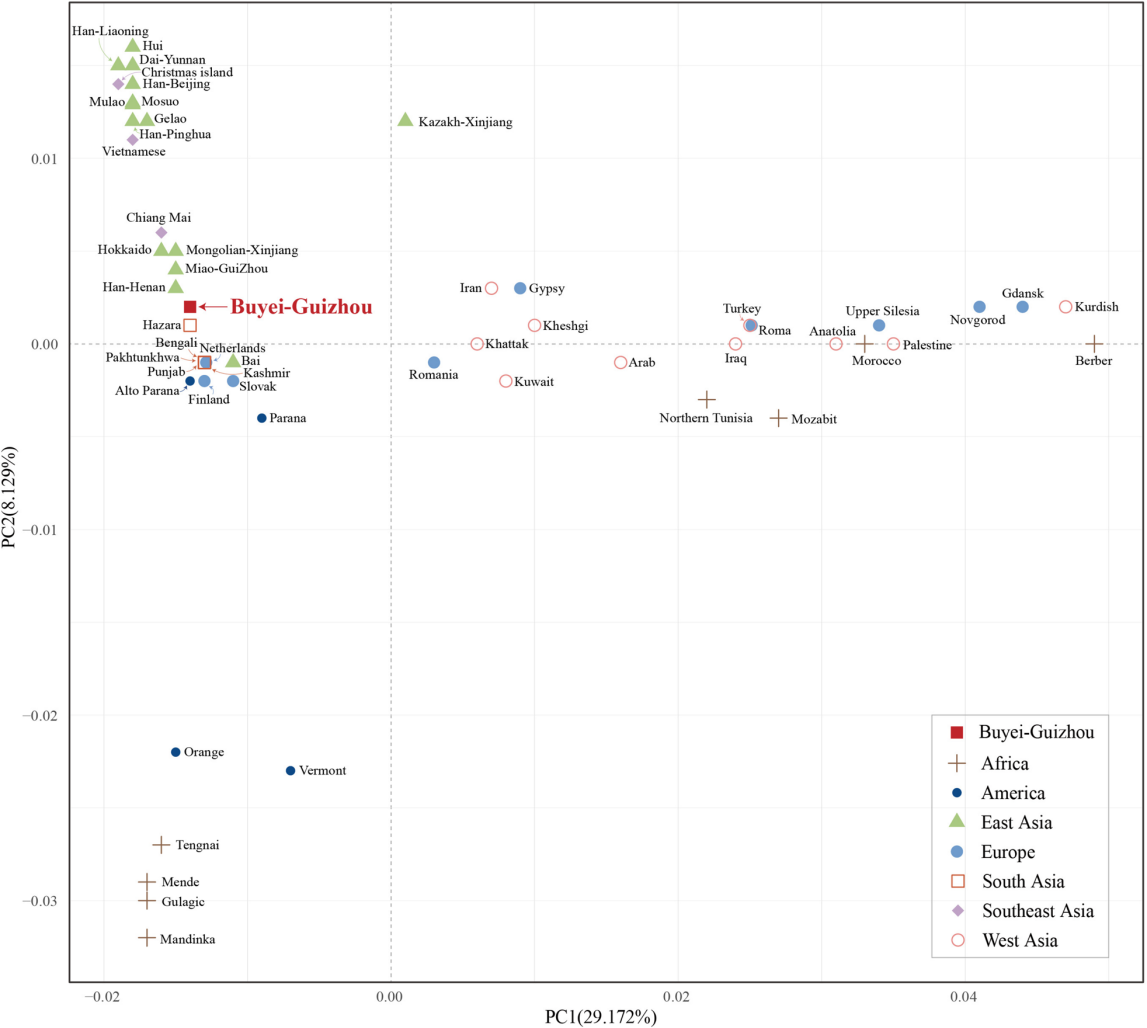
The PCA plot for the Guizhou Buyei population and 53 worldwide populations according to geographic distributions. The research population belongs to the East Asia cluster (indicated in the green triangle). The Buyei-Guizhou was highlighted in bold red and indicated by arrows

To elucidate the genetic relationship between the Buyei population of Guizhou and global populations, the PCA based on haplogroup frequencies was also conducted. The results of the PCA based on haplogroup frequencies indicated that the first three principal components account for 43.488% of the variation. Specifically, PC1 explains 29.172% of the variation, PC2 accounts for 8.129%, and PC3 for 6.187%. The PCA visualisation (Fig. 2) showed that geographic clustering reveals two distinct clusters: one made up of African populations and the other of mixed East and Southeast Asian populations. The plot revealed that West Asian populations and certain European populations cluster together, with instances of partial overlap observed. Through PC1 and PC2, most populations can be differentiated; however, these components have virtually no effect on European populations. The detailed findings indicated that the point representing the Buyei population of Guizhou is situated within the East Asian cluster, in close proximity to the points representing the Han Chinese from Henan (Han-Henan) as well as the Hazaras of Pakistan (Hazara).

**Fig. 3:**
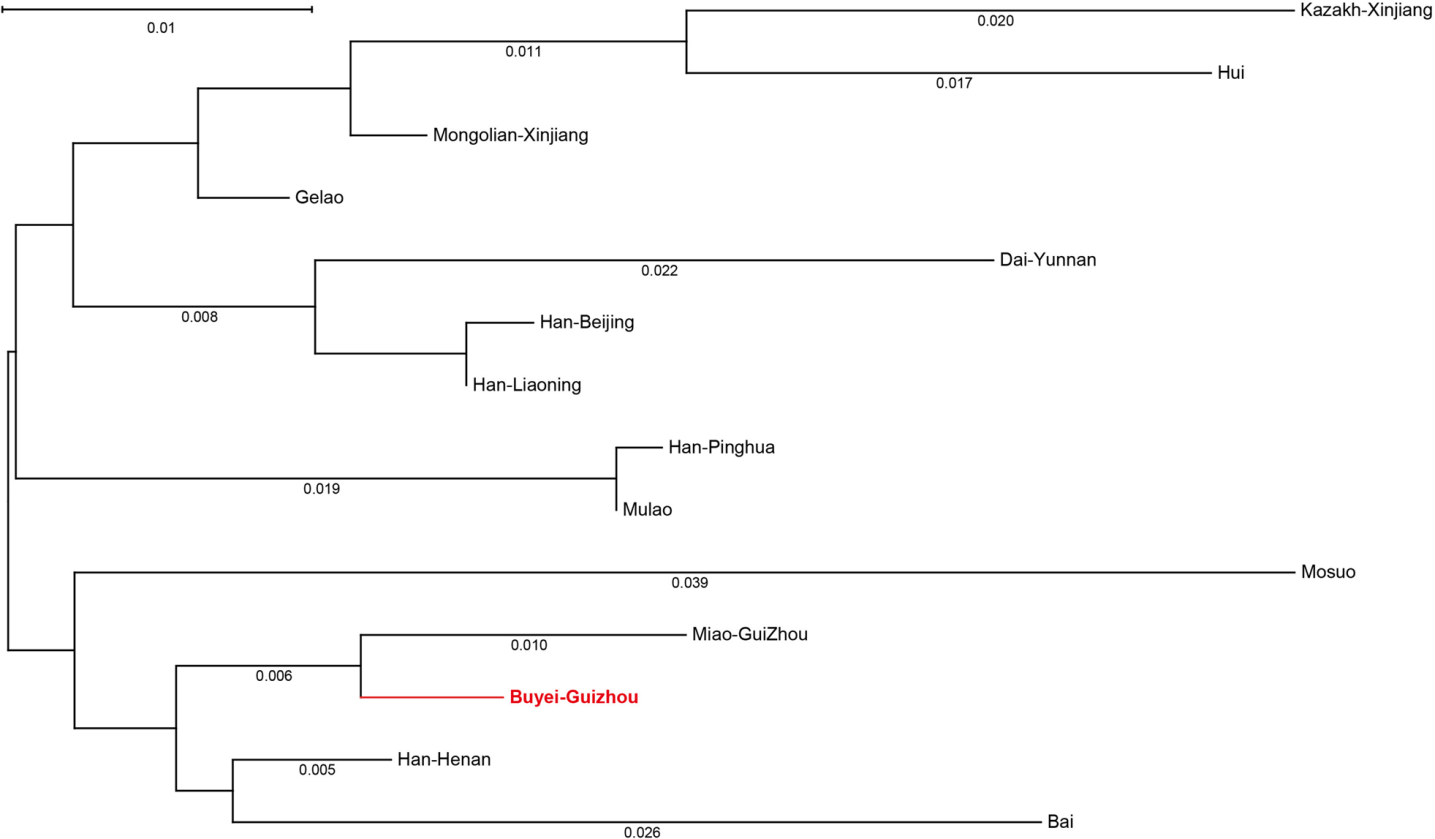
The NJ phylogenetic tree based on the paired *Fst* distance matrix between Guizhou Buyei and 13 Chinese populations. Highlight the research population of Buyei-Guizhou with bold red text

**Fig. 4:**
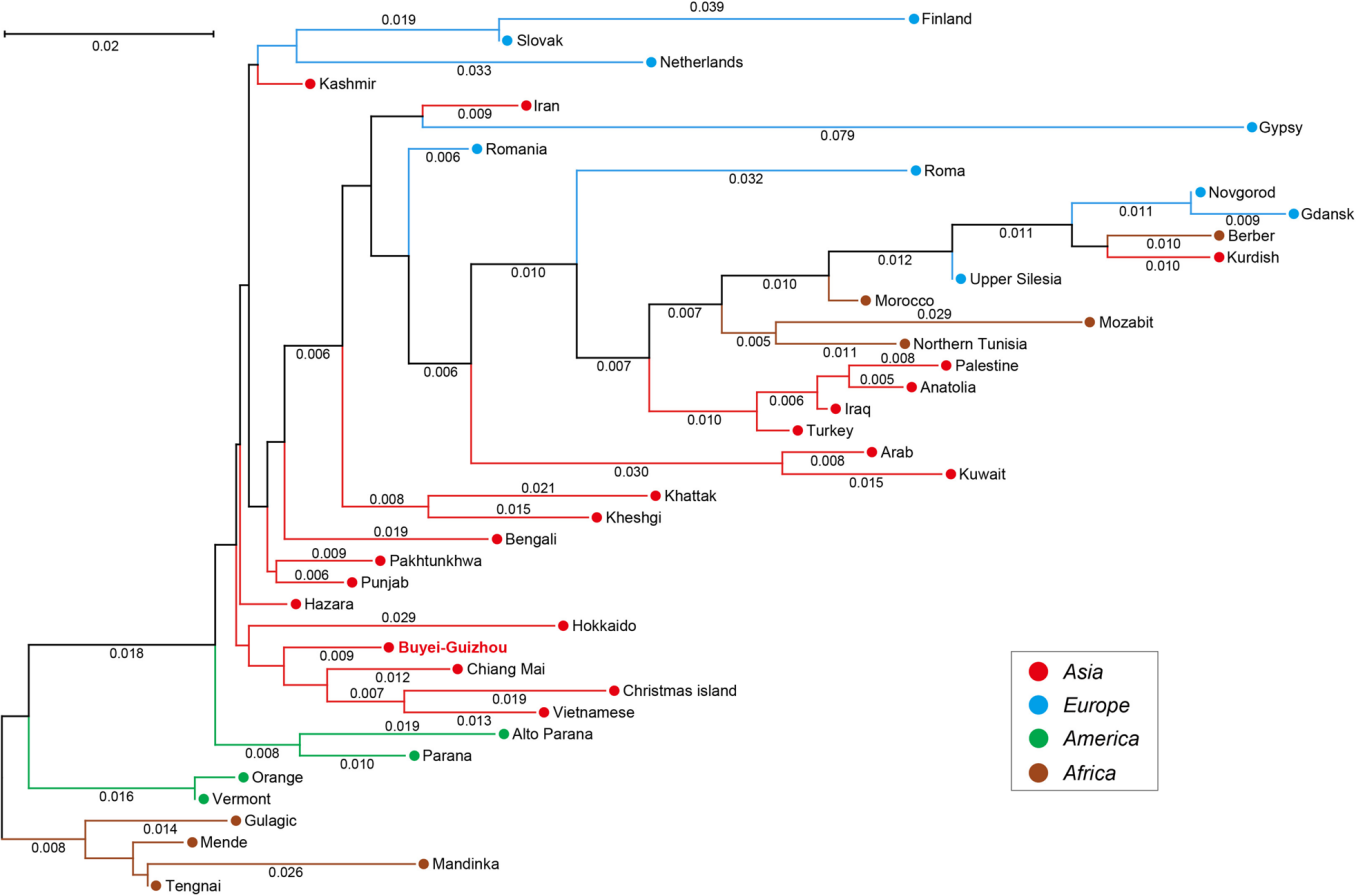
The NJ phylogenetic tree based on the paired *Fst* distance matrix between Guizhou Buyei and 40 populations in the world. Group populations according to the different continents and mark each population with distinct colours

To elucidate the phylogenetic relationships between the Guizhou Buyei and global reference populations, an NJ phylogenetic tree was constructed based on *Fst* values (Figs. 3 and 4). The NJ tree, based on the pairwise *Fst* genetic distances among the research population and 13 reference populations within China, as depicted in Fig. 3, indicated that the Guizhou Buyei clusters on the same branch as the Miao population, which is also located in Guizhou. Additionally, the research population shared a relatively close phylogenetic relationship with the Han population from Henan. This showed that the genetic distance differentiation among the three populations is comparatively small, which is consistent with what is shown in Table S2. Based on the NJ tree, which has the Buyei population of Guizhou and 40 reference populations from around the world (excluding China, Fig. 4), the results showed that the research population is grouped with other Asian populations in a main branch, with the Tai-Kadai-speaking population in Chiang Mai, Thailand (Chiang Mai), having the closest phylogenetic relationship.

Through the analysis of the *Fst* genetic matrix, PCA, and NJ tree, it could be concluded that geographic location, historical factors, and language families have a certain influence on gene intergenerational transmission. Based on the characteristics of matrilineal inheritance, the genetic distance of mtDNA haplogroups might be positively correlated with the geographical distance and language families among populations.

## Discussion

In this research, the haplotypes and haplogroups of mtDNA of the Buyei population in Guizhou were studied and analysed, and it could be found that many individuals had the same haplotype. MtDNA is matrilineal, so the selected samples may come from the same matrilineal line, although this point could not be confirmed by their ID cards or oral descriptions. In addition, it might also be because the polymorphism of the mtDNA control region is not sufficient to distinguish these individuals.

The results of the population genetics analysis indicated that the Guizhou Buyei has a very close genetic relationship with the Miao population in the same region, which might be related to their geographical location and common genetic pattern. Moreover, due to the differences in language and culture, there was little gene communication between ethnic minorities and Han nationalities, which led to a great differentiation of genetic distance among them. Nevertheless, in our study, there was little genetic differentiation between Henan Han and Guizhou Buyei. This might be related to the origin and history of the Buyei population and the historical status of Henan. According to historical documents, the ancestors of the Buyei people were one of the main ethnic groups in the ancient Yelang Kingdom, but after the development of this country, its control area was far beyond the areas inhabited by the Buyei ancestors, and many tribes might live in these areas [50]. During this period, gene exchanges between the Buyei nationality and the Han nationality might take place. In addition, research has recorded that in the Ming and Qing dynasties, the court dispatched a large number of Han troops into Guizhou to hoard the military, and many soldiers married local Buyei people and merged into the Buyei. It showed that the Buyei ethnic group may have absorbed a small part of the Han ethnicity in the Ming and Qing dynasties [51]. And Henan belongs to the Yellow River basin, one of the birthplaces of the ancient Han population [52]. Although the gene exchange between the modern Han nationality and other ethnic groups was extremely rare, under the above historical background, the gene exchange between the ancient Han nationality and ethnic minorities was possible. Due to the matrilineal inheritance of mitochondria, it has been accumulated so far, which is consistent with the results of our study. The above discussion was only supported by historical data and wasn’t supported by scientific data. Consequently, we still need to rely on modern science and technology for further research and verification, such as mitochondrial whole genome sequencing and so on.

On the other hand, compared with other populations in the world (except China), the Guizhou Buyei exhibited a closer genetic affinity with populations in Asia, likely due to geographical proximity. Notably, the studied population shared close genetic relationships with South Asian populations, particularly the Hazara people of Pakistan, which may stem from Pakistan’s historical role in ancient trade and commerce as well as the presence of the ancient “Silk Road” facilitating genetic exchanges [53]. This was very consistent with the effect of geographic location and historical background factors on genetic differentiation. The close relationship between the Guizhou Buyei and the people of Chiang Mai, Thailand, is likely influenced by their geographical location, and both populations belong to the Tai-Kadai linguistic family.

## Conclusion

To sum up, in our study, haplotypes, haplogroups, and the population structure of the Buyei population in Guizhou were analysed based on mtDNA. The sequences of the mtDNA control region of the Buyei nationality in Guizhou had high polymorphism and a large amount of information. It could provide detailed information about the degree of mtDNA variation and haplogroup distribution. Additionally, it could be widely used in forensic case studies, anthropological analysis, and population genetics research. In addition, through the analysis of genetic polymorphism and population structure, it was found that the genetic distance of the Guizhou Miao, Henan Han, Hazara, and Chiang Mai populations were close to that of the Guizhou Buyei population. It was found that the genetic relationship between the Guizhou Buyei and other reference populations was predominantly consistent with how they have spread geographically and linguistically. This means that the maternal lineages determined by mtDNA exhibit a close correlation with geographical factors and linguistic families.

### Supplementary Information

**Additional file 1: Table S1.** Haplotypes observed in 200 Buyei individuals. In 200 individuals, a total of 179 haplotypes (89.5%) were observed, of which 163 haplotypes were unique (91.1%).

**Additional file 2:** **Table S2.** The pairwise *Fst* values (below diagonal lines) and *p*-values (above diagonal lines) of Guizhou Buyei population and 53 other published populations worldwide. The paired *Fst* and *p*-values of the Guizhou Buyei population and 13 other published populations in China were calculated. The genetic differentiation between the Guizhou Buyei and Guizhou Miao was the smallest (with the closest genetic affinity, *Fs*t= 0.01508), followed by the Henan Han nationality (*Fst*= 0.01799). The genetic distance between the northwest Hui and Guizhou Buyei was the largest (with the farthest genetic affinity, *Fs*t= 0.05908).The paired *Fst* genetic distance and correlation coefficient p-values between Guizhou Buyei nationality and 40 other reference populations in the world (except China) showed that Guizhou Buyei nationality and Pakistan Hazara nationality had the smallest genetic distance (with the closest genetic affinity, *Fst*= 0.01783), followed by Kashmiri (*Fst*= 0.02084), and had the largest genetic differentiation (with the farthest genetic affinity, *Fst*= 0.12165) with the Gdansk people in Poland.

**Additional file 3: Figure S1. **Guizhou Buyei were assigned to 89 different haplogroups and sub-haplogroups. The most common haplogroups were B5a (18 cases, 9%), followed by M7b1a1+(16192) (17 cases, 8.5%), B4b1 (7 cases, 3.5%), and R9 (14 cases, 7%).

## Data Availability

The mitochondrial DNA control region sequences of all Tai-Kadai-speaking Buyei individuals generated in this study have been submitted to GenBank (https://www.ncbi.nlm.nih.gov/genbank/), and the accession number was ON983171-ON983370.
