# Supplementary material for: Analysis of maternal genetic structure of mitochondrial DNA control region from Tai-Kadai-speaking Buyei population in southwestern China
Source: BMC Genomics. 2024 Jan 11;25:50. doi: 10.1186/s12864-023-09941-x (PMC10782584; doi:10.1186/s12864-023-09941-x)
Supplement: Supplementary file 3 — Additional file 3: Figure S1. Guizhou Buyei were assigned to 89 different haplogroups and sub-haplogroups. The most common haplogroups were B5a (18 cases, 9%), followed by M7b1a1+(16192) (17 cases, 8.5%), B4b1 (7 cases, 3.5%), and R9 (14 cases, 7%). [file 12864_2023_9941_MOESM3_ESM.doc]

**
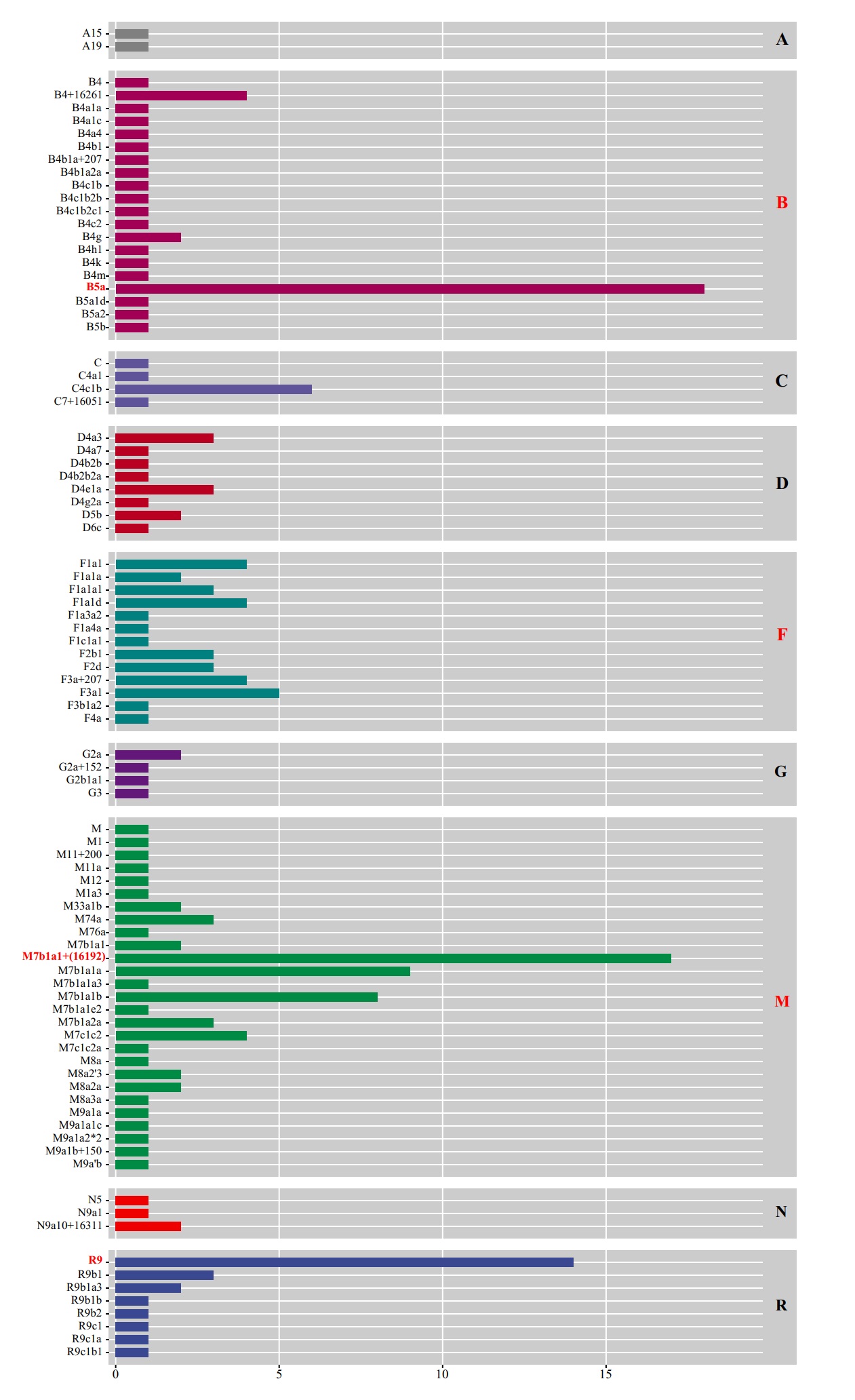
**

**Figure S1. Guizhou Buyei were assigned to 89 different haplogroups and sub-haplogroups.** The most common haplogroups were B5a (18 cases, 9%), followed by M7b1a1+(16192) (17 cases, 8.5%), B4b1 (7 cases, 3.5%), and R9 (14 cases, 7%).
